# The Effects of Intermittent Cold Exposure on Adipose Tissue

**DOI:** 10.3390/ijms25010046

**Published:** 2023-12-19

**Authors:** Matthew C. Scott, Scott Fuller

**Affiliations:** 1 Pennington Biomedical Research Center, Baton Rouge, LA 70808, USA; matthew.scott@pbrc.edu; 2 School of Kinesiology, University of Louisiana at Lafayette, Lafayette, LA 70506, USA

**Keywords:** intermittent cold exposure, ICE, cold exposure, adipose tissue, brown adipose tissue, beiging

## Abstract

Intermittent cold exposure (ICE) has garnered increased attention in popular culture, largely for its proposed effects on mood and immune function, but there are also suggestions that the energy-wasting mechanisms associated with thermogenesis may decrease body weight and fat mass. Considering the continued and worsening prevalence of obesity and type II diabetes, any protocol that can reduce body weight and/or improve metabolic health would be a substantial boon. Here, we present a narrative review exploring the research related to ICE and adipose tissue. Any publicly available original research examining the effects of repeated bouts of ICE on adipose-related outcomes was included. While ICE does not consistently lower body weight or fat mass, there does seem to be evidence for ICE as a positive modulator of the metabolic consequences of obesity, such as glucose tolerance and insulin signaling. Further, ICE consistently increases the activity of brown adipose tissue (BAT) and transitions white adipose tissue to a phenotype more in line with BAT. Lastly, the combined effects of ICE and exercise do not seem to provide any additional benefit, at least when exercising during ICE bouts. The majority of the current literature on ICE is based on rodent models where animals are housed in cold rooms, which does not reflect protocols likely to be implemented in humans such as cold water immersion. Future research could specifically characterize ICE via cold water immersion in combination with controlled calorie intake to clearly determine the effects of ICE as it would be implemented in humans looking to lower their body weight via reductions in fat mass.

## 1. Introduction

Obesity and type 2 diabetes constitute a global epidemic. The dysfunction of adipose tissue has been implicated in the pathophysiology of metabolic syndrome (MetS) [1], which is a cluster of symptoms including abdominal obesity, impaired blood glucose homeostasis, hypertension, and dyslipidemia [2]. MetS is an underlying factor in heart disease, stroke [3], and cognitive impairments such as Alzheimer’s disease and dementia [4]. Properly functioning adipose tissue is indispensable in the maintenance of metabolic health, as has been established in numerous studies in animals and humans [5]. 

Given the irreplaceable role of healthy adipose tissue in maintaining proper metabolic status, intense investigation has focused on enhancing the function of adipose tissue in several contexts. As an example, one class of drugs, thiazolidinediones (TZDs), has demonstrated evidence of improving glucose homeostasis via its action on adipocytes via the induction of PPARγ, the master transcriptional regulator of adipocyte function [6]. However, TZDs exhibit undesirable side effects including weight gain, cardiac complications, and increased bone fractures [7]; therefore, they are generally contraindicated. In view of the paucity of favorable outcomes in treating obesity and adipose tissue dysfunction with pharmacological approaches, strategies utilizing lifestyle modification such as dietary and exercise interventions have been explored as logically understandable methods for promoting weight loss, maintenance, and combating metabolic abnormalities. However, despite decades of admonitions to reduce caloric intake and increase energy expenditure using these traditional approaches, the obesity epidemic has proven to be recalcitrant and has only intensified in recent years [8].

Recently, the practice of intermittent cold exposure (ICE) has received attention in popular culture as a modality for improving mood, increasing immune function, and reducing inflammation, with some scientific evidence backing those anecdotal reports [9,10,11,12]. ICE has also been suggested as a potential modulator of body weight and adipose tissue function. Cold exposure challenges the maintenance of thermal homeostasis, to which homeothermic animals must mount an orchestrated set of defensive responses. These include sympathetic nervous system activation, shivering, and non-shivering thermogenesis [13]. The activation of brown adipose tissue (BAT), which specializes in high rates of substrate metabolism, thereby generating heat as a by-product, is a critical mechanism by which core body temperature is maintained in response to cold exposure [14]. Given the capacity of BAT to metabolize substrate at high rates per unit of tissue mass, it is logical to hypothesize that the activation, and perhaps expansion, of BAT might constitute a modality to combat obesity by increasing energy expenditure (EE). Indeed, support for this notion is available in the literature [15,16]. Perhaps more exciting is the speculation that white adipose tissue (WAT), which generally does not exhibit high rates of substrate oxidation, might undergo a shift toward a phenotype more consistent with BAT, a phenomenon referred to as “beiging” or “browning”. By activating BAT and altering the metabolic function of WAT, cold exposure might exert specific effects on adipose tissue that could act therapeutically in the treatment of obesity and its associated pathologies [17,18].

This narrative review aims to summarize the current state of the field pertaining to intermittent cold exposure as an intervention to combat obesity and obesity-induced metabolic syndrome. Emphasis will be placed on the specific effects of repeated bouts of ICE on adipose tissue. The review will discuss the mechanisms by which ICE could alter the metabolic and endocrine function of adipose tissue in a manner consistent with increased resistance to obesity, thereby attenuating the disease burden stemming from the dysfunction of adipose tissue, which constitutes a hallmark characteristic of metabolic syndrome.

## 2. Overview of Included Literature

We have included all the available literature that examined the effects of intermittent cold exposure (ICE) on adipose tissue or, at the very least, body weight. The methods varied and are described both here and in Table 1 with specific detail given to the ICE protocols, although many of the studies had broader interests, which are partially described in the group columns of Table 1. In all, we have 23 studies that have spanned from 1979 to 2023; however, the data presented in Deshaies et al. are essentially an addendum to Arnold et al., where both studies are reporting outcomes from the same animal cohort [19,20]. The majority of the included literature examined the effects of ICE on rodents with a fairly even mix of rat and mouse models, although literature examining the effects of ICE on adipose tissue in humans is also present. There was quite a bit of variability in the ICE protocols with respect to the ICE duration (<5 min–8 h) and the span of intervention (2 weeks–6 months). Further, the rodent studies exclusively used cold rooms (air) while studies in humans used a combination of air, water-cooled suits, cold showers, and cold water immersion. Cold showers and cold water immersion would be the most likely methods for implementation for people looking to adopt an ICE protocol, but the majority of the literature, again, comprises rodents being housed in cold rooms. The ICE frequency in our included studies was fairly consistent; most studies examined ICE at 5 days per week or daily. Lastly, most studies examine an ICE intensity at 4–10 °C, but some utilized temperatures as low as −20 °C for a shorter duration.

## 3. ICE Effects on Body Weight, Energy Expenditure, and Adipose Tissue

The recent increased interest in intermittent cold exposure (ICE) is related to the proposed benefits for mood, inflammation, immune function, and general physical wellness. While not as commonly mentioned, there are suggestions that ICE could reduce body weight as a function of the increased energy expenditure (EE) due to shivering (ST) and non-shivering thermogenesis (NST). ST and NST are considered the major energy sinks in the context of cold exposure [40,41], but there is the potential for other factors related to ICE to modulate energy homeostasis, such as hormone modulation [42,43], that are not discussed in this section. Here, and in Table 2, we outline the effects of ICE on body weight (BW), adipose tissue weight (white adipose tissue (WAT) and brown adipose tissue (BAT), adipose tissue morphology (WAT and BAT), EE, and outcomes that may be related to EE.

### 3.1. Body Weight and Energy Expenditure

The effects of ICE on BW are mixed, with studies reporting increases [21,25,33,38], decreases [19,22,37], and null effects [9,24,27,29,30,35]. Still, the methodologies were varied, and a closer look provides some potential insights. Studies using human subjects generally show no change in BW [9,35] but do report a modest decrease in waist circumference [9], while rodent studies represent much of the variability initially mentioned. ICE duration was examined directly by Ravussin et al. and Tsibul’nikov et al. The former directly measured the differential effects of ICE at 1, 4, or 8 h/d and all conditions demonstrated no change in BW [27]. The latter compared 1.5 h to 8 h of ICE, where both groups demonstrated an increased BW, and the increase in BW was more substantial with the shorter ICE duration [33]. The intensity of ICE may add additional context as Harri et al. used −20 °C for 60 min per ICE session and found a significant decrease in weight gain over the course of the study [22]. Arnold et al. also used a comparatively more intense ICE temperature, −5 °C, where a decreased BW was also reported [19]. Lastly, the length of ICE interventions did not serve as an explanatory factor, where longer interventions, 10–14 wks, seem to confirm a null effect [27,29] and shorter interventions cover the gamut of the initially mentioned disparity. The overall data on ICE do not provide solid evidence on modulating body weight in either direction but increases in intensity may be more likely to induce a reduction in BW.

The inconsistency is less dramatic as related to EE in response to ICE. Studies generally indicate increased energy expenditure [16,19,36], further increasing with increased ICE duration [27]. While EE is clearly increased in response to ICE, Presby et al. adds additional nuance, examining EE in the light and dark cycles. ICE was shown to induce increased EE during the times generally associated with sleep for rodents, but decreased EE during the active dark cycle [36]. However, Presby et al. implemented ICE 3 h prior to the sleep cycle, which may implicate the ICE timing rather than ICE generally [36].

An overall null effect of ICE on BW is expected when the balance between the EE and energy intake (EI) is maintained, and an expected increase in EI, as an effect of ICE, is reported by most studies [19,23,27,37,38]. Interestingly, Yoo et al. capped the EI to pre-ICE intervention levels and still reported an increase in BW [25]. Unfortunately, they reported limitations in their ability to accurately measure the total EE in the ICE mice, instead reporting no difference in the EE at thermoneutrality in both the control and ICE mice without account for their EE during CE. Further, Presby et al. also controlled the EI and reported a null effect on BW along with an overall null effect on the EE, despite an increased EE during cold exposure [36]. Still, when considering the combined reports, ICE seems to increase both the EE and EI when calories are available ad libitum, resulting in an overall null effect on BW based on the combined literature.

### 3.2. White Adipose Tissue Weight and Morphology

Despite the lacking evidence for ICE to modulate BW, there is some evidence for its impact on white adipose tissue (WAT) weight and implications for WAT function based on morphology. Generally, ICE tends to increase the subcutaneous WAT (sWAT) [25,32,38,39] and has variable effects on the visceral WAT (vWAT), where increases [25,27,30,38], decreases [20,23,24,29,32], and null effects [36,39] are reported. In humans, winter swimmers have an increased fat mass, but a decrease in vWAT as estimated via electrical impedance [32]. However, Yoneshiro et. al. reports that 6 weeks of ICE, via cold air, was sufficient to induce modest decreases in fat mass [24]. In rodents, Yoo et al. reports an increase in both sWAT and vWAT [25], a report that is not consistent across the ICE-related literature. As mentioned above, Yoo et al. maintained the EI to pre-ICE intervention levels, but still report a significant increase in WAT weight, which accounts for most of their reported increases in BW [25]. However, they also showed that ICE induced an increase in WAT multilocularity, with multiple lipid droplets within a single adipocyte. This suggests a transition to a more metabolically active beige phenotype of WAT and is indicative of an increased thermogenic capacity [44]. The report of ICE-induced WAT beiging is consistent with other morphology-based reports [30,36]. Overall, the reports for changes in WAT mass are too inconsistent to be conclusive, but the sWAT tends to increase in response to repeated bouts of ICE. Further, studies support ICE as a protocol for inducing a beige adipocyte morphology, related to increased mitochondria and thermogenesis, which may indicate improvements in the metabolic activity of the WAT in rodents and humans exposed to ICE. More support for ICE-induced WAT beiging is discussed in Section 4.

### 3.3. Brown Adipose Tissue Weight, Morphology, and Energy Expenditure

While WAT beiging is associated with an increased capacity for WAT thermogenesis, brown adipose tissue (BAT) is generally considered the prominent site for adipocyte thermogenesis per tissue weight, although the WAT does comprise a substantially larger tissue mass, which seems to increase with ICE. ICE tends to increase BAT weight, based on the majority of studies [22,29,33,35,36]. However, one study indicates no change [27] and another reports a decrease [23]. While there is a small disparity in the reports for ICE-induced increases in BAT weight, there are clear effects on BAT activity. ICE consistently increased the thermogenic response of BAT to a cold stress, where rodents [25] and humans [16,24,31,34] that undergo regular ICE show increased BAT activation in response to cold exposure. Interestingly, BAT activation is decreased at thermoneutrality in humans who participate in ICE compared to those who do not [16], which may be related to Hanssen et al.’s report of a decreased basal metabolic rate [31]. Of particular note, Yoneshiro et al. specifically examined the effects of ICE on individuals with low or non-detectable BAT activity at baseline. They noted that 50% of the individuals with non-detectable BAT activity had measurable activity after 6 wks of regular ICE [24]. Overall, ICE increases BAT activation in response to cold stress and likely increases BAT weight.

### 3.4. Overall Conclusions from Table 2

While ICE increases EE, BAT activation, and WAT beiging, there is no clear evidence for its potential to decrease FM and BW. In fact, some studies report both increased BW and FM [25,32,38], which may implicate compensatory mechanisms based on the consensus for ICE to increase EI. While BW and FM may increase, the increase seems to be predominantly in the sWAT rather than vWAT, where increases in the vWAT would be generally associated with poor metabolic and health outcomes. Further, both the sWAT and vWAT consistently adopt a beiging phenotype in response to ICE, and a transition from a primarily energy storing phenotype to a more metabolically active tissue could imply additional benefits. There may still be possibility for ICE-induced weight loss when combined with caloric restriction, although reports from EI-controlled studies suggest a substantial caloric deficit would be needed [25,36].

## 4. ICE Effects on Adipose Tissue Gene and Protein Expression

Adipose tissue function, both in a tissue mass and secretory/endocrine context, is modulated by alterations in gene and protein expression in response to environmental stimuli. Intermittent cold exposure (ICE) induces a potent challenge to thermal homeostasis, thereby evoking systemic responses mobilized to defend core body temperature. Such systemic responses represent a complex array of interactions between gene and protein expression in cold-responsive tissues and circulating factors that results in a network of crosstalk underlying the physiological responses to ICE. For example, a recent review summarized an interesting set of responses to increased glucagon, which some evidence indicates can act as a thermogenic factor and as a browning activator, largely via its action as a stimulator of FGF21 secretion [45]. Adipose tissue has been demonstrated in several studies to be highly responsive to ICE. This responsiveness is not limited to the brown adipose tissue (BAT), although BAT has been regarded, logically, to be a major player in regulating body temperature via its high rates of substrate turnover, enabled by abundant mitochondria and the high expression of uncoupling proteins (UCPs). These UCPs enable high rates of substrate oxidation uncoupled from generating ATP, with the result of this uncoupled cellular respiration being robust heat generation, and therefore maintenance of homeostatic body temperature in response to cold challenge. However, research interest in ICE’s effect on adipose tissue has expanded beyond the relatively well-understood role of BAT in cold tolerance to the examination of how ICE could alter the patterns of gene and protein expression in the white adipose tissue (WAT). Changes in adipose tissue gene and protein expression were reported by 11 of our 20 included studies. Those results are presented in Table 3 and discussed below.

### 4.1. Brown Adipose Tissue Gene and Protein Expression

BAT is characterized by abundant mitochondria, high expression of UCP1, and multilocular lipid droplets, underlying many of the adaptive thermogenic responses to cold exposure and enabling the defense of body temperature in euthermic animals. The cellular lineage of BAT is markedly different from WAT and exhibits similarity to skeletal muscle insofar as both cell types derive from stem cell precursors expressing the transcription factors Pax7 and Myf5 [46]. The responsiveness of BAT to cold exposure has logically received abundant attention due to its specialized capacity to generate heat by dissipating chemical energy in the mitochondria via uncoupled cellular respiration and ATP synthesis. A line of experimental evidence has supported the overall theme that BAT responds to cold exposure by increasing the tissue mass via hypertrophy and hyperplasia, the induction of uncoupling protein 1 (UCP1) and PPARG coactivator 1 alpha (PGC1α) expression, and increasing the fat utilization capacity via the increased expression of the lipoprotein lipase [27,29,36].

### 4.2. White Adipose Tissue Gene and Protein Expression

WAT has traditionally been regarded solely as a somewhat passive site for the storage of excess caloric energy in the form of neutral lipids, thus enabling this tissue to serve as a regulator of nutrient homeostasis [47]. However, upon the discovery of adipocyte-derived factors such as leptin and adiponectin, scientific interest in adipose tissue as a bona fide endocrine organ intensified greatly [48]. WAT has been shown to be highly responsive to temperature; indeed, subcutaneous WAT is uniquely situated to provide sensory input on ambient temperature to organisms [47].

Experiments on mice examining the responsiveness of WAT, in the subcutaneous and visceral fat depots, to cold exposure provide evidence that a thermogenic transcriptional program was induced in a cell autonomous manner [49]. The notion that WAT could shift toward a phenotype more related to BAT and thereby become more thermogenic is tantalizing insofar as such a shift could result in greater energy expenditure, representing a possible anti-obesity therapeutic modality. Several experiments have demonstrated that ICE results in alterations specifically in WAT gene and protein expression that underlie a thermogenic transcriptional program. Increased UCP1 expression in WAT has been demonstrated in several studies, supporting the notion that cold exposure could result in increased substrate oxidation in WAT [26,29,36], supporting the notion for the ICE-induced beiging of white adipose tissue.

Interestingly, beige or “brite” (brown within white) adipocytes have been shown to constitute a distinct cell type resident within WAT depots exhibiting high responsiveness to cold exposure and ready induction of a thermogenic gene expression program enabled by sensitivity to irisin, a polypeptide hormone secreted by muscle [44,50]. In this context, a mechanism by which ICE could act as an exercise mimetic can be proposed, thereby making this concept more concrete. Further support for the notion that WAT is highly plastic and sensitive to temperature was provided in experiments showing that the thermogenic capacity of beige adipocytes induced by cold exposure within WAT depots can be reversed upon the removal of cold exposure for a duration of 5 weeks [51].

### 4.3. Overall Conclusions from Table 3

Taken together, results mainly from rodent experiments support the notion that ICE promotes the initiation of a thermogenic gene expression profile in both BAT and WAT, with a more robust induction of thermogenesis in BAT than in WAT. This is expected given that BAT is specialized, particularly in rodents, to generate heat by dissipating the normally tight coupling between substrate oxidation and ATP synthesis. Regardless, the confirmation that WAT can undergo beiging upon exposure to cold challenge implicates this tissue as a potentially thermogenic factor. While evidence that cold exposure can independently result in the loss of body weight and reduce the body fat percentage is lacking, the shift from a primarily energy-storing to an energy-dissipating phenotype offers a rationale for considering ICE as a modality to promote metabolic health insofar as this shift in gene and protein expression could result in greater energy expenditure. In this context, alterations in adipose tissue gene and protein expression promoting thermogenesis constitute a line of evidence supporting the use of ICE as a modality to promote a beneficial metabolic phenotype that is resistant to the development of insulin resistance and the metabolic consequences of obesity.

## 5. ICE Effects on Systemic Factors Related to Adipose Tissue and Metabolism

### Insulin, Glucose Homeostasis, and Adipokines

Metabolic syndrome (MetS) is characterized by defects in insulin action and blood glucose homeostasis stemming from hyperinsulinemia and insulin resistance in the peripheral tissue [52]. Moreover, evidence from multiple studies has implicated hyperinsulinemia per se as a hallmark abnormality underlying a leading cause of mortality globally, atherosclerotic cardiovascular disease [53]. Given the role played by insulin resistance and hyperglycemia in morbidity and mortality, interventions showing potential in enhancing insulin action in the peripheral tissue present opportunities to combat a range of pathologies associated with metabolic syndrome. In view of the indispensable role played by healthy adipocytes in maintaining metabolic health via their endocrine signaling and the evidence for cold exposure in favorably altering the metabolic function of adipose tissue [29,47], it is reasonable to speculate that intermittent cold exposure (ICE) could enhance insulin sensitivity and glucose homeostasis via specific effects on adipose tissue. In Table 4, we summarize the reports for ICE-induced changes in these systemic factors and discuss them below.

Numerous studies have investigated the effect of cold exposure on the secretory function of adipose tissue and the possible modulation of insulin sensitivity and blood glucose via changes in circulating adipokines such as adiponectin and leptin. Unfortunately, the effectiveness of ICE in inducing these systemic factors remains ambiguous, with no clear consensus emerging from the studies undertaken to date. The evidence for ICE as a potent stimulus for the increased secretion of adiponectin and leptin, which promote insulin sensitivity and satiety, respectively, is somewhat limited. While Ravussin et al. observed a transient increase in glucose tolerance without direct effects on insulin in mice, there was no increase in serum adiponectin nor were there changes in body weight; these observations were largely attributed to compensatory increases in food intake in response to ICE [27]. This logically leads one to hypothesize that the administration of supplementary leptin in ICE might offset compensatory increases in food intake. Unexpectedly, Mckie et al. show the null effect of leptin injection in mice undergoing an ICE protocol but shows the expected reduction in food intake for control mice [38]. With respect to adiponectin, it is notable that adipose-tissue-specific adiponectin KO mice did not exhibit the same extent of adaptation to ICE as control mice, perhaps indicating that adiponectin plays at least a permissive role in mediating adaptations to ICE, thereby underscoring the importance of adipose tissue as a mediator in response to ICE [26].

Although direct evidence of a linkage between ICE and the modulation of adipokines, such as leptin and adiponectin, is fairly limited, some studies have demonstrated favorable alterations in glucose homeostasis and insulin sensitivity associated with changes in adipose tissue biochemical factors. Weng et al. reported that a combination of ICE and exercise in rats resulted in several effects on free fatty acid (FFA) production and utilization, producing the net effect of reducing insulin resistance, HOMA-IR, and blood glucose, with a main effect of ICE independent of exercise [39]. This study further demonstrated that adipose triglyceride lipase (ATGL) and lipoprotein lipase (LPL) activity in inguinal adipose tissue were increased in response to ICE and that the skeletal muscle FFA oxidative capacity elevated via increases in PPARG coactivator 1 alpha (PGC-1α) and p38 mitogen-activated protein kinases (p38MAPK) in response to ICE, thereby offsetting the increases in FFA delivered to the circulation via increased lipolysis. Further evidence for ICE in promoting insulin sensitivity and glucose homeostasis has been provided by trials in rodent and human subjects. For example, Hanssen and colleagues found that 10 days of exposure to ambient temperatures of 14–15 °C increased the insulin sensitivity and basal GLUT-4 translocation in skeletal muscle, in males with type II diabetes [31]. Although the literature is currently limited, human trials have demonstrated a pattern of decreased fasting glucose and increased insulin sensitivity assessed using glucose tolerance tests in response to ICE [16,32].

## 6. Effects of Exercise during ICE

Health-conscious individuals implementing intermittent cold exposure (ICE) are also likely to include other lifestyle practices to improve physical function. Three of our included ICE studies also examined the effects of exercise and ICE in combination, so we have reported those effects in Table 5 and discuss them here. Again, however, the reports from Arnold et al. and Deshaies et al. originate from the same cohort of animals [19,20]. Arnold and Deshaies report a decreased body weight (BW) in response to ICE alone [19,20]. While Weng et al. do not report BW, they do note a trend for increased subcutaneous white adipose tissue (sWAT) [39], where altogether the variable effects of ICE on BW and fat mass are preserved in relation to the larger body of literature. Exercise was shown to reduce BW [19,20] but resulted in no change in sWAT when sWAT trended higher with ICE alone [39]. In all studies, the combination of ICE and exercise did not provide any additional benefit with respect to reduced body weight or sWAT. More generally, the combination of exercise and ICE blunts the effect of ICE alone, where the combination provides the same benefit of exercise alone. Unfortunately, all three studies examined the effects of exercise during the CE associated with ICE, and it is likely that the heat generated during exercise simply blunted the thermogenic stress associated with ICE. There may be increased potential to derive a combinatory benefit of exercise and ICE if the exercise bout is implemented outside of the ICE bout.

## 7. Conclusions and Future Directions

The global obesity and type 2 diabetes epidemics constitute a grave threat to public health and demand effective interventions. Efforts to develop pharmaceuticals and decades of generic advice on lifestyle modification (i.e., “eat less, move more”) have thus far not succeeded in favorably altering the global trajectory toward ever-increasing rates of obesity and type 2 diabetes. Given the magnitude of the healthcare and economic burdens associated with metabolic dysfunction, the need for effective prevention and treatment has never been more acute.

Intermittent cold exposure (ICE), due to its effects on energy expenditure (EE), seems a logical avenue of approach in combating obesity and its associated pathologies, as cold exposure induces a metabolic energy demand. While ICE has received attention in the context of adipose tissue biology related to its effect on brown adipose tissue (BAT) thermogenesis, recent experimental evidence indicates that white adipose tissue (WAT) also engages a set of adaptive responses to cold challenge by becoming more thermogenic, a phenomenon referred to as “beiging” [44]. These adaptive responses at the level of gene expression provide a mechanistic basis for understanding how changes in WAT could underlie alterations in the total energy expenditure and energy intake. With respect to our review of the ICE-related literature, ICE clearly increases BAT activity and generally increases BAT mass. Further, ICE certainly transitions WAT to a beige phenotype based both on the morphology and molecular signaling, but also seems to promote increased adiposity. As a mechanism for weight loss, the evidence does not support ICE; however, the trend for improved metabolic outcomes in response to ICE may still indicate its potential as an anti-diabetic intervention.

The above conclusions are based on sparse literature in human subjects, where the majority of research examines rodent models housed in cold rooms. In our opinion, there is a need for an expansion of scientific investigation into the modalities of ICE most likely to be implemented by humans. The most likely option would be cold water immersion, which provides a much more intense bout of cold exposure with a reduced duration. This is not to say that all future research should utilize human subjects, but that the effects of ICE via cold water immersion would likely provide the most relevant outcomes in relation to what would most likely be implemented. Lastly, relevant investigations of cold water immersion, in humans or rodents, would provide a methodological basis for future research aimed at parsing the effects of ICE intensity, duration, and frequency, similar to the literature on other lifestyle modifications such as exercise.

## Figures and Tables

**Table 1:** ICE methods.

Year	First Author	Citation	Methods						
			Model	Groups	ICE Modality	ICE Freq.	ICE Intensity	ICE Duration	ICE Intervention
1979	Doi	[21]	Rats	1. Control Newborn 2. Control Adult 3. ICE Newborn 4. ICE Adult	Air	daily	5 °C	4 h	14 days
1984	Harri	[22]	Rats	1. Control 2. CCE 3. ICE 4. Control Exercise 5. CCE Exercise	Air	5 d/wk	−20 °C	1 h	≥6 wks
1986	Arnold	[19]	Rats	1. Control 2. ICE 3. Control Exercise 4. ICE Exercise	Air	daily	−5 °C	2 h	28 days
1988	Deshaies	[20]	Rats	1. Control 2. ICE 3. Control Exercise 4. ICE Exercise	Air	daily	−5 °C	2 h	28 days
1989	Yahata	[23]	Rats	1. Control 2. CCE 3. ICE	Air	daily	5 °C	6 h or 2 h	28 days
2013	Yoneshiro	[24]	Humans (Low or No BAT Activity During Initial ACE)	1. Control Pre vs. Post 2. ICE Pre vs. Post	Air	daily	17 °C	2 h	6 wks
2014	Yoo	[25]	Mice	1. Control 2. ACE 3. ICE	Air	daily	4 °C	6 h	12–14 days
2014	Qiao	[26]	Mice	1. Control ICE 2. Adipoq^−/−^ ICE	Air	daily	4 °C	6 h	10 days
2014	Ravussin	[27]	Mice	1. Control 2. ICE 1 h 3. ICE 4 h 4. ICE 8 h	Air	3 d/wk	4 °C	1, 4, or 8 h	10–11 wks
2014	Blondin	[28]	Humans	Pre vs. Post ICE	Liquid-cooled suit	5 d/wk	18 °C	2 h	4 wks
2015	Wang	[29]	Mice	1. Control 2. ICE	Air	5 d/wk	4 °C	2 h	14 wks
2015	Bai	[30]	Platue Pika (Rodent) Simulated Low Air Pressure (4100 M)	1. Control 2. ICE	Air	daily	5–6 °C	4 h	15 days
2015	Hanssen	[31]	Humans (Obese)	Pre vs. Post ICE	Air	daily	15 °C	6 h	10 days
2016	Gibas-Dorna	[32]	Humans	1. Controls 2. Winter Swimmers (Pre vs. Post)	Winter swimming	≥2 d/wk	1–10 °C	5–15 min	~6 months
2016	Tsibul’nikov	[33]	Rats	1. Control 2. CCE 3. ICE (1.5 h) 4. ICE (8 h)	Air	daily	4 °C	1.5 or 8 h	4 wks
2016	Hanssen	[34]	Humans (Overweight, T2DM)	Pre vs. Post ICE	Air	daily	15 °C	6 h	10 days
2017	Blondin	[35]	Humans	Pre vs. Post ICE	Liquid-cooled suit	daily	10 °C	2 h	4 wks
2019	Presby	[36]	Obese Mice Caloric Restriction During ICE 24 h ad libitum after ICE	1. Caloric Restriction 2. ICE + Caloric Restriction	Air	5 d/wk	4 °C	1.5 h	3 wks
2021	Soberg	[16]	Humans	1. Controls 2. Winter Swimmers	Winter swimming	2–3 d/wk	NA	11 min	At least 1 season
2021	Zhang	[37]	Rats	1. Control 2. ICE	Air	daily	10 °C	4 h	2 wks
2022	McKie	[38]	Obese Mice	1. HFD 2. ICE + HFD	Air	5 d/wk	4 °C	1 h	4 wks
2023	Weng	[39]	Obese Rats	1. Control 2. Exercise 3. ACE 4. ACE + Exercise 5. CCE 6. CCE + Exercise 7. ICE 8. ICE + Exercise	Air	daily	3–4 °C	4 h	5 wks
2023	Nema	[9]	Humans (Soldiers)	1. Control Pre vs. Post 2. ICE Pre vs. Post	CWI and CS	CWI ≥ 1/wk CS ≥ 4/wk	CWI ≤ 6 °C CS ≤ 10 °C	CWI ≥3 min CS ≥ 30 s	8 wks

Legend: chronic cold exposure (CCE), intermittent cold exposure (ICE), acute cold exposure (ACE), cold water immersion (CWI), cold shower (CS), not applicable (NA), high-fat diet (HFD).

**Table 2:** ICE effects on body weight, adipose tissue weight/morphology, and energy balance.

Year	First Author	Citation	ICE Outcomes	Model
1979	Doi	[21]	↑BW (adults), ↔BW (newborns), ↑BAT, ↑EE in response to NorEp, ↑NST, ↓ST	Rats
1984	Harri	[22]	↓Weight gain, ↔BW (trended ↓), ↑BAT, ↑NST, ↓ST	Rats
1986	Arnold	[19]	↓BW, ↓FFM, ↓FM, ↑BAT, ↑EE, ↑EI	Rats
1988	Deshaies	[20]	↓BW, ↓eWAT and %eWAT, ↑BAT and %BAT	Rats
1989	Yahata	[23]	↓BW, ↓eWAT, ↓BAT, ↑EI	Rats
2013	Yoneshiro	[24]	↔BW, ↓FM, ↔FFM. ↑BAT activity during CE, ↑EE during CE	Humans (No BAT Activity During Initial ACE)
2014	Yoo	[25]	↑BW, ↔FFM, ↑FM, ↑iWAT, ↑iWAT adipocyte size, ↑iWAT beiging, ↑eWAT, ↓eWAT adipocyte size, ↑BAT, ↔EE	Mice
2014	Qiao	[26]	Adipoq^−/−^: ↑iWAT beiging, ↓thermogenesis	Mice
2014	Ravussin	[27]	Cohort 1 (1 or 4 h ICE): ↔BW, ↔FM, ↔FFM, ↔iWAT, ↔eWAT, ↔BAT, ↑EE (4 h), ↑EI (4 h) Cohort 2 (4 or 8h ICE): ↔BW, ↔FM, ↔FFM, ↔iWAT, ↑eWAT (4 & 8 h), ↔BAT, ↑EE (4 h), ↑↑EE (8 h), ↑EI (4 h), ↑↑EI (8 h)	Mice
2014	Blondin	[28]	↑BAT activity during CE, ↓skin temp, ↓ST	Humans
2015	Wang	[29]	↔BW, ↔sWAT, ↓sWAT adipocyte size, ↓vWAT, ↑BAT activity, ↑BAT adipocyte number	Mice
2015	Bai	[30]	↔BW, ↔sWAT, ↓sWAT adipocyte size, ↑pericardial WAT, ↓pericardial adipocyte size, ↑pericardial WAT beiging	Platue Pika (Rodent)
2015	Hanssen	[31]	↑BAT activity during CE, ↑EE during CE, ↓basal metabolic rate at thermoneutrality	Humans (Overweight,T2D, Males)
2016	Gibas-Dorna	[32]	Winter swimmers vs. controls: ↑BW, ↑FM, ↓vWAT Winter swimmers (pre vs. post winter swimming season): ↔BW, ↔FM, ↔vWAT	Humans
2016	Tsibul’nikov	[33]	↑BW (↑8 h, ↑↑1.5 h), ↑BAT weight (8 h)	Rats
2016	Hanssen	[34]	↑BAT activity during CE	Humans (Obese)
2017	Blondin	[35]	↔BW, ↑BAT volume, ↑BAT activity with CE, ↓skin temp, ↓ST, ↑EE with CE, ↔fuel utilization during CE	Humans
2019	Presby	[36]	↔BW, ↔FM, ↔FFM, ↔iWAT, ↔eWAT, ↑sWAT beiging, ↑BAT weight, ↑BAT adipocyte size, ↑EE during CE, ↓EE during dark cycle, ↑EE during light cycle	Obese Mice Caloric Restriction During ICE 24 h ad libitum after ICE
2021	Soberg	[16]	Winter swimmers: higher supraclavicular skin temp in response to cold exposure, no BAT glucose uptake at thermoneutrality (controls had glucose uptake at thermoneutrality), ↑glucose uptake in perirenal BAT during cold exposure (not significant for control), ↑REE during cold exposure	Humans
2021	Zhang	[37]	↓BW, ↑EI (trend)	Rats
2022	McKie	[38]	↑BW, ↑iWAT, ↑eWAT, ↑BAT, ↑EI, ↑↑EI (within 4 h post ICE)	Obese Mice
2023	Weng	[39]	↑sWAT, ↔vWAT	Obese Rats
2023	Nema	[9]	↔BW, ↔BMI, ↔FM, ↔FFM, ↓Waist Circumference (men only)	Humans (Soldiers)

Legend: increased (↑) (↑↑), no change (↔), decreased (↓), cold exposure (CE), intermittent cold exposure (ICE), body weight (BW), fat mass (FM), fat-free mass (FFM), white adipose tissue (WAT), inguinal WAT (iWAT), epididymal WAT (eWAT), subcutaneous WAT (sWAT), visceral WAT (vWAT), brown adipose tissue (BAT), non-shivering thermogenesis (NST), shivering thermogenesis (ST), energy expenditure (EE), energy intake (EI).

**Table 3:** ICE effects on adipose genes and proteins.

Year	First Author	Citation	ICE Outcomes	Model
1984	Harri	[22]	↑Citrate synthase in BAT, ↑cytochrome C oxidase in BAT	Rats
1988	Deshaies	[20]	↔eWAT LPL	Rats
1989	Yahata	[23]	↑BAT glucagon	Rats
2014	Yoo	[25]	↑iWAT *Ucp1* and UCP1, ↑BAT UCP1, ↑eWAT lipogenic gene expression (*Scd1*, *Lpl*, *Pparγ)*	Mice
2014	Qiao	[26]	Adipoq^−/−^: ↑iWAT UCP1	Mice
2014	Ravussin	[27]	Cohort 1 (1 or 4 h ICE): ↑BAT *Ucp1* (4 h), ↑BAT *Pgc1α* (4 h), ↓serum leptin (4 h) Cohort 2 (4 or 8 h ICE): ↑BAT UCP1 (4 & 8 h)	Mice
2015	Wang	[29]	↑sWAT UCP1 and PGC1α, ↔BAT UCP1 (trended↑)	Mice
2015	Bai	[30]	↑BAT thermogenic gene expression (*Pgc1α*, *Dio2*, *Cidea*) and adipogenic gene expression (*Adipoq*, *Cebpα*, *Pparγ*, *Fabp4*), ↑pericardial WAT thermogenic gene expression (*Ucp1* and *Pgc1α*), ↑pericardial WAT UCP1 staining and mito activity genes (*Cox8* and *ATP5α*), ↔serum leptin	Platue Pika (Rodent)
2019	Presby	[36]	↑sWAT *Dio2*, ↑sWAT UCP1, ↑BAT *Dio2*, ↑BAT UCP1	Obese Mice Caloric Restriction During ICE 24 h ad libitum after ICE
2021	Zhang	[37]	↑peri-ovarian adipose thermogenic gene expression (*Ucp1*, *PGC1α*, *Prdm16*, *Fndc5*)	Rats
2023	Weng	[39]	↔iWAT ATGL activity, ↑iWAT LPL activity	Obese Rats

Legend: increased (↑), no change (↔), decreased (↓), cold exposure (CE), intermittent cold exposure (ICE), white adipose tissue (WAT), epididymal WAT (eWAT), inguinal WAT (iWAT), subcutaneous WAT (sWAT), brown adipose tissue (BAT), messenger ribonucleic acid (mRNA), steroyl-CoA desaturase 1 (*Scd1*, mRNA), lipoprotein lipase (LPL, protein; *Lpl*, mRNA), peroxisome proliferator-activated receptor gamma (*Pparγ*, mRNA), uncoupling protein 1 (UCP1, protein; *Ucp1*, mRNA), PPARG coactivator 1 alpha (PGC1α, protein; *Pgc1α*, mRNA), iodothyronine deiodinase 2 (*Dio2*, mRNA), cell-death-inducing DFFA like effector a (*Cidea*, mRNA), adiponectin (*Adipoq*, mRNA), CCAAT-enhancer-binding protein alpha (*Cebpα*, mRNA), fatty-acid-binding protein 4 (*Fabp4*, mRNA), cytochrome c oxidase subunit 8A (*Cox8*, mRNA), ATP synthase F1 subunit alpha (*Atp5α*, mRNA), PR domain-containing 16 (*Prdm16*, mRNA), fibronectin type III domain-containing protein 5 (Fndc5, mRNA), adipose triglyceride lipase (ATGL, protein).

**Table 4:** ICE effects on systemic factors.

Year	First Author	Citation	ICE Outcomes	Model
1988	Deshaies	[20]	↓serum cholesterol, ↔HDL	Rats
1989	Yahata	[23]	↑plasma glucagon, ↑corticosterone	Rats
2014	Yoo	[25]	↔IS, ↑liver TGs and hepatic TG release, ↑DNL	Mice
2014	Ravussin	[27]	cohort 1 (1 or 4 h ICE): ↑BAT glucose uptake cohort 2 (4 or 8 h ICE): ↑FFA (4 & 8 h), ↓insulin	Mice
2014	Blondin	[28]	↓BG, ↓cortisol	Humans
2015	Wang	[29]	↑glucose tolerance, ↑insulin sensitivity	Mice
2015	Bai	[30]	↔serum glucose, ↔serum TG	Platue Pika (Rodent)
2015	Hanssen	[31]	↑IS, ↑GLUT4 translocation in skeletal muscle	Humans (Overweight,T2D, Males)
2016	Gibas-Dorna	[32]	↑IS during the portion of the winter swimming season where water temperature was <8 °C	Humans
2016	Hanssen	[34]	↑skeletal muscle glucose uptake during ICE, ↑GLUT4 translocation in skeletal muscle	Humans (Obese)
2017	Blondin	[35]	↔FG	Humans
2021	Soberg	[16]	winter swimmers had ↓plasma glucose during IGTT	Humans
2022	McKie	[38]	↑glucose tolerance, ↑insulin and c-peptide in response to glucose, ↔IS	Obese Mice
2023	Weng	[39]	↔BG, ↔insulin, ↓HOMA-IR, ↔FFA	Obese Rats

Legend: increased (↑), no change (↔), decreased (↓), intermittent cold exposure (ICE), brown adipose tissue (BAT), high density lipoproteins (HDL), insulin sensitivity (IS), triglyceride (TG), de novo lipogenesis (DNL), blood glucose (BG), fasting glucose (FG), intravenous glucose tolerance test (IGTT), homeostatic model assessment for insulin resistance (HOMA-IR), free fatty acid (FFA).

**Table 5:** Combined effects of ICE and exercise.

Year	First Author	Citation	Intervention	Outcomes	Model
1986	Arnold	[19]	ICE	↓BW, ↓FFM, ↓FM, ↑BAT, ↑EE, ↑EI	Rats
			Ex	↓↓BW, ↓FFM, ↓BAT, ↓↓FM, ↔EE, ↓EI	
			ICE + Ex	↓↓BW, ↓FFM, ↓↓FM, ↔BAT, ↓EI, ↔EE	
1988	Deshaies	[20]	ICE	↓BW, ↓eWAT, ↑BAT, ↑EI, ↔TG, ↓Cholesterol	Rats
			Ex	↓↓BW, ↓↓eWAT, ↔BAT, ↓EI, ↓TG, ↔Cholesterol	
			ICE + Ex	↓↓BW, ↓↓eWAT, ↔BAT, ↓EI, ↓TG, ↔Cholesterol	
2023	Weng	[39]	ICE	↔sWAT (trend↑), ↑sWAT LPL activity, ↑muscle PGC1α, ↑muscle p38MAPK	Obese Rats
			Ex	↔sWAT, ↑sWAT LPL activity, ↑muscle PGC1α, ↑muscle p38MAPK	
			ICE + Ex	↔sWAT, ↑↑sWAT LPL activity	

Legend: increased (↑), significantly increased compared to ↑ (↑↑), no change (↔), decreased (↓), significantly decreased compared to ↓ (↓↓), treadmill running exercise (Ex), Ex during ICE (ICE + Ex), intermittent cold exposure (ICE), body weight (BW), fat mass (FM), fat-free mass (FFM), epididymal WAT (eWAT), subcutaneous WAT (sWAT), brown adipose tissue (BAT), energy expenditure (EE), energy intake (EI), triglyceride (TG), lipoprotein lipase (LPL), PPARG coactivator 1 alpha (PGC1α, protein), mammalian p38 mitogen-activated protein kinase (p38MAPK, protein).
